# Optimising chemical information workflows: integrating Reaxys - use cases and applications

**DOI:** 10.1186/1758-2946-5-S1-P39

**Published:** 2013-03-22

**Authors:** Sebastian Radestock

**Affiliations:** 1 Elsevier Information Systems GmbH, Frankfurt am Main, 60486, Germany

## 

Reaxys is an authoritative web-based workflow solution designed for chemistry researchers in drug discovery, chemicals and academic research [1]. Reaxys supports research and fuels discovery by integrating searches for reaction and substance data with synthesis planning and chemical sourcing.

With historical coverage dating right back to 1771 and continuing through todays cutting-edge research, Reaxys covers the most important chemistry-related literature and patent sources and provides unparalleled depth of information on reactions, substances and related property data.

To make the most effective use of this information Reaxys offers two new entry points into the data:

• Chemical space analysis, analogue sourcing, fuelling hit-to-lead programs and IP prior-art searching are possible through the Reaxys Structure Flat File.

• Programmatic interaction with the database by custom clients, downloading bulk data from Reaxys using scripts, executing batch queries and integrating Reaxys into server-based data-intensive pipelining or workflow applications are possible through the Reaxys APIs.

The Reaxys Structure Flat File and the Reaxys APIs will facilitate chemoinformaticians and information managers to integrate Reaxys into chemical information processing environments. In our poster, we present common use cases and possible applications. In addition, we describe successful implementations, illustrating how additional insights and efficiency can be gained.
